# Multiscale studies on the nonlinear vibration of delaminated composite laminates–global vibration mode with micro buckles on the interfaces

**DOI:** 10.1038/s41598-017-04570-3

**Published:** 2017-06-30

**Authors:** Jianghong Xue, Fei Xia, Jun Ye, Jianwen Zhang, Shuhua Chen, Ying Xiong, Zuyuan Tan, Renhuai Liu, Hong Yuan

**Affiliations:** 1 Institute of Applied Mechanics, School of Mechanics and Construction Engineering, Jinan University, Guangzhou, Guangdong 510632 China; 2 Key Lab of Disaster Forecast and Control in Engineering, Ministry of Education, Guangzhou, Guangdong 510632 China; 3 Department of Statistics, The University of Akron, Akron, OH 44303 USA

## Abstract

This paper presents a multiscale approach to study the nonlinear vibration of fiber reinforced composite laminates containing an embedded, through-width delamination dividing the laminate into four sub-laminates. The equations of motion are established from macroscopic nonlinear mechanics for plates and shells and micro-mechanics of composite material to allow for the influences of large amplitude, membrane stretching in the neutral plane, and the interactions of the sublaminates. Analytical solutions obtained in this paper reveal that the interaction penalty at the interfaces plays a coupling effect between sublaminates, which eventually alters the vibration characters of the four-sublaminate lamina in macroscopic and microscopic mechanism. From a macro perspective, sub-laminates above and below the delamination vibrate in exactly the same mode in spite of their different stiffness and the four-sublaminate lamina has a consistent global vibration mode. In accompanying with the macro vibration, micro buckles occur on the interfaces of the delamination with amplitude about 10^−3^ times of that of the global mode. It is found that the vibration frequency is an eigenvalue of the delaminated lamina determined only by the geometry of the delamination. Authentication of the multiscale study is fulfilled by comparing the analytical solutions with the FEA results.

## Introduction

Carbon-fiber-reinforced composite materials consist of two parts: matrices and reinforcements. The reinforcements are usually carbon fibers which provide the strength and rigidity for the composite materials, measured by the stress and the elastic modulus respectively. The matrix is usually a polymer resin, such as epoxy, to bind the reinforcements together. Unlike isotropic materials, such as steel and aluminum, carbon-fiber-reinforced composite has directional strength and properties. The properties of carbon-fiber-reinforced composite depend on the layouts of the carbon fiber and the proportion of the carbon fibers in the polymer.

During manufacturing process, fiber breakage, matrix cracking, and fiber/matrix interface debonding may exist in the composite materials and, as a result, develop delamination in the composite materials. Delamination is one of the primary failure types in composite laminates and will cause the stiffness degradation and strength loss of the entire laminate, consequentially the premature failure of the composite materials. The failure of fiber reinforced composite structures containing buried delamination is much complex due to presence of various mechanisms. The analysis of the response of the delaminated composite structures under the external loadings needs to account for various factors, e.g., large deformation, nonlinear constitutive behavior, failure, delamination, and interaction penalty effects, etc. Ignoring the interaction penalty effects may result in the underestimation of the load carrying capacity and the penetration of the deflection between the sublaminates above and below the interfaces of delamination^1^. Therefore, researchers have launched various studies to investigate the effects of contact on the mechanical responses of the delaminated composite structures.

Numerous approaches as well as different results have been reported in many literatures. Chai *et al*.^2^ may be one of the first to carry out the study of the delaminated composite laminates. By simplifying the composite plate as a beam-plate, they proposed a one dimensional analytical model to assess the compressive strength of a delaminated composites plate. Cranford^3^ investigated the mechanical stability of graphene-based nanoscale multi-layers and derived the critical length scale and required adhesion energy to prevent delamination under buckling conditions. Oyewole *et al*.^4^ presented the results of experimental and theoretical/computational micro-wrinkles and buckling on the surfaces of stretchable poly-dimethylsiloxane (PDMS) coated with nano-scale Gold (Au) layers. The interfacial delamination occurred along with the buckling was also studied using finite element simulations of the interfacial crack growth. Ovesy *et al*.^5^ suggested a novel layerwise theory to evaluate the buckling and post-buckling behavior of delaminated composite plates with multiple through-the-width delaminations based on the first order shear deformation theory (FSDT). Giannakopoulos *et al*.^6^ and Whitcomb^7^ investigated the stability by considering the nonlinearity associated with the contact problem. The results reveal that the effects of the contact conditions are significant, especially with regard to the propagation of the debonded region. Hu *et al*.^8^ introduced artificial springs to compute the fictitious contact forces occurred in the buckling of laminates with an embedded delamination based on the Mindlin plate theory. The other research topics include simulation of the multi-failure responses, see Ho *et al*.^9^; multiscale method to compute the elastic deformations for general bilayer, see Kumar *et al*.^10^; contact of interfacial debonding front region and face matrix cracking, etc. in face/core interface debonded composite sandwich plates, see Chen and Bai^11^; the non-linear behaviors of delaminated sandwich panels with a compressible core, see Frostig and Thomsen^12^; fatigue delamination growth for piezoelectric laminated cylindrical shells, see Zhu *et al*.^13^; analysis of the mixed mode delamination in composite laminates from point view of fracture mechanics and contact mechanics, see Bruno *et al*.^14^; experimental studies on laminated plates with strip-type delamination under pure bending, see Yet *et al*.^15, 16^.

Except the static analysis, the dynamic responses of composite laminate with delamination are also the focus of a number of studies. One of the earliest models for vibration analysis of composite beams with delaminations was proposed by Ramkumar *et al*.^17^. They modeled a beam with one through-width delamination by simply using four Timoshenko beams connected at delamination edges. Wang *et al*.^18^ improved the analytical solution by including the coupling between flexural and axial vibrations of the delaminated beams which were modeled as split regions. Recurrence equations relating integration constants for adjacent split regions were established by satisfying continuity conditions at junctions of sub-regions. Ramkumar *et al*.’s and Wang *et al*.’s models were essentially one-dimensional models. Dynamic responses of laminates with two-dimensional embedded delamination were studied by Dey and Karmakar^19^ to examine free vibration of multiple delaminated angle-ply composite conical shells using Mindlin’s theory and the multi-point constraint algorithm and by Noh and Lee^20^ to analyze the dynamic stability of laminated skew plates by developing a finite element formulation based on HSDT. Such models are called “free model”. As pointed out in present published paper^21^, the “free model” result in interpenetration of the delaminated sub-regions, which is physical impossible. To overcome this defect a piecewise linear spring model inserted in the delaminated region was introduced. Several researchers conducted their studies based on this model. Luo and Hanagud^22^ carried out the dynamic response analysis of a delaminated beam by taking into account of shear effect, rotary inertia terms and bending-extension coupling. Kargarnovin *et al*.^23^ examined a delaminated Timoshenko beam under the motion of a constant amplitude point force traveling with uniform velocity by accounting for the Poisson’s effect, shear deformation and rotary inertia. Chen *et al*.^24, 25^ presented a formula of element stiffness and mass matrices for the composite laminates using the first-order shear deformation theory combined with the selecting numerical integration scheme. A virtual interface linear spring element was employed to avoid the overlap and penetration phenomenon between the upper and lower sublaminates at the delamination region. Chattopadhyay *et al*.^26^ presented an FE model using the first-order zig–zag theory for delaminated composites and smart composite plates. The linear spring model needs to specify the spring constant before solving the problem, which is basically equivalent to specifying whether the sublaminates are in contact or not beforehand, this model therefore does not reflect the essential feature of the contact problem, that is, the contact region and the degree of interpenetration are all unknown a priori. Oh *et al*.^27^ and Kwon and Aygunes^28^ developed finite element models adopting the approach given by Hughes *et al*.^29^ for correcting the velocity, acceleration and contact force values during release-to-contact condition. Their models were able to achieve nonpenetration of interlaminates but the results they present can still be argued upon for its physical correctness, as both the top and bottom laminates experience a kind of total separation. In reality, they must collide more than once during the vibration as both of the sub-laminates are shown to be vibrating by themselves at higher frequencies than the contact occurrence. Recently, Wang and Tong^30^ suggested a nonlinear interpenetration constraint model to handle the contact problem. In this nonlinear model, the contact force was considered to be proportional to the relative deflections of the mid-planes of subregions above and below the delamination interfaces. Schwarts-Givli *et al*.^31^ further put forward a step function in the nonlinear constrain model to incorporate “with and without contact” conditions in the governing equations, which reflects the nonlinear nature of the contact behavior.

Although, a vast literature exists on the effects of delamination on the dynamic response of composite structures, they have mostly ignored large deformation effects. When delaminated laminates undergo large deflection, the large deflection induces geometric nonlinearity in the delaminated region and leads to the couple effect between the membrane stretching and transverse bending and the occurrence of the contact phenomenon at the delaminated region. As a result, non-uniform interaction constrains are produced along the interfaces in-between the upper and lower sublaminates. They restrict the two sublaminates to deform penetrating with each other by causing a local compressive stress and a vertical deformation along the interfaces of the delamination^32–34^. The bending analysis of the delaminated plates is an interdiscipline and multiscale problem. The research involves two mechanical mechanisms: theories of plates and interaction mechanics. The multiscale composition of the composite lamina causes it to undergo macro deformation as well as micro buckling. The problem becomes even more intricate due to the obscurity of the interaction properties, such as the locations and sizes of the contact area, the intensity of the contact force, etc. For this reason, almost all the analyses and results in above mentioned literatures are accomplished in virtue of computer techniques. Such analyses may lead to an argument that the contact properties, such as the locations and the sizes of the contact area, are unpredictable.

In this article, we develop a general framework to analyze the nonlinear vibration of fiber-reinforced composite laminate with embedded delamination using a multi-scale modeling approach and infer possible consequences of delamination geometry on vibration properties. While the interaction forces at the interfaces of the delamination is investigated from the point view of micro-mechanics, governing equations for the delaminated laminates undergoing nonlinear vibration are derived based on von Karman nonlinear mechanics for plates and shells to include nonlinear geometry deformation and membrane stretching and are solved via Galerkin approach. To demonstrate the validity of our method, we analyze the nonlinear free vibration of a simply supported, symmetrically cross-plied laminate with a though-width delamination. We obtain close-form analytical solutions and provide the macroscopic and microscopic explanation for the outcome of the consistent macro vibration mode and the micro buckles on the interfaces of the delamination. We have compared the vibration frequency as well as the vibration mode predicted by our multi-scale approach with the dynamics simulations from ABAQUS, obtained an excellent agreement between these two approaches.

## Theoretical modelling

### Material modeling

This article mainly focuses on investigating the nonlinear vibration of a delaminated composite laminate. The study includes investigation of the overall dynamic responses of the composite laminate and determination of the local transverse deformation of sub-laminates above and below the delamination due to the interaction forces between them. The overall dynamic responses of the composite laminate are mainly achieved by virtue of shell theories in which the in-plane material properties is of much more significance.

**Figure 1:**
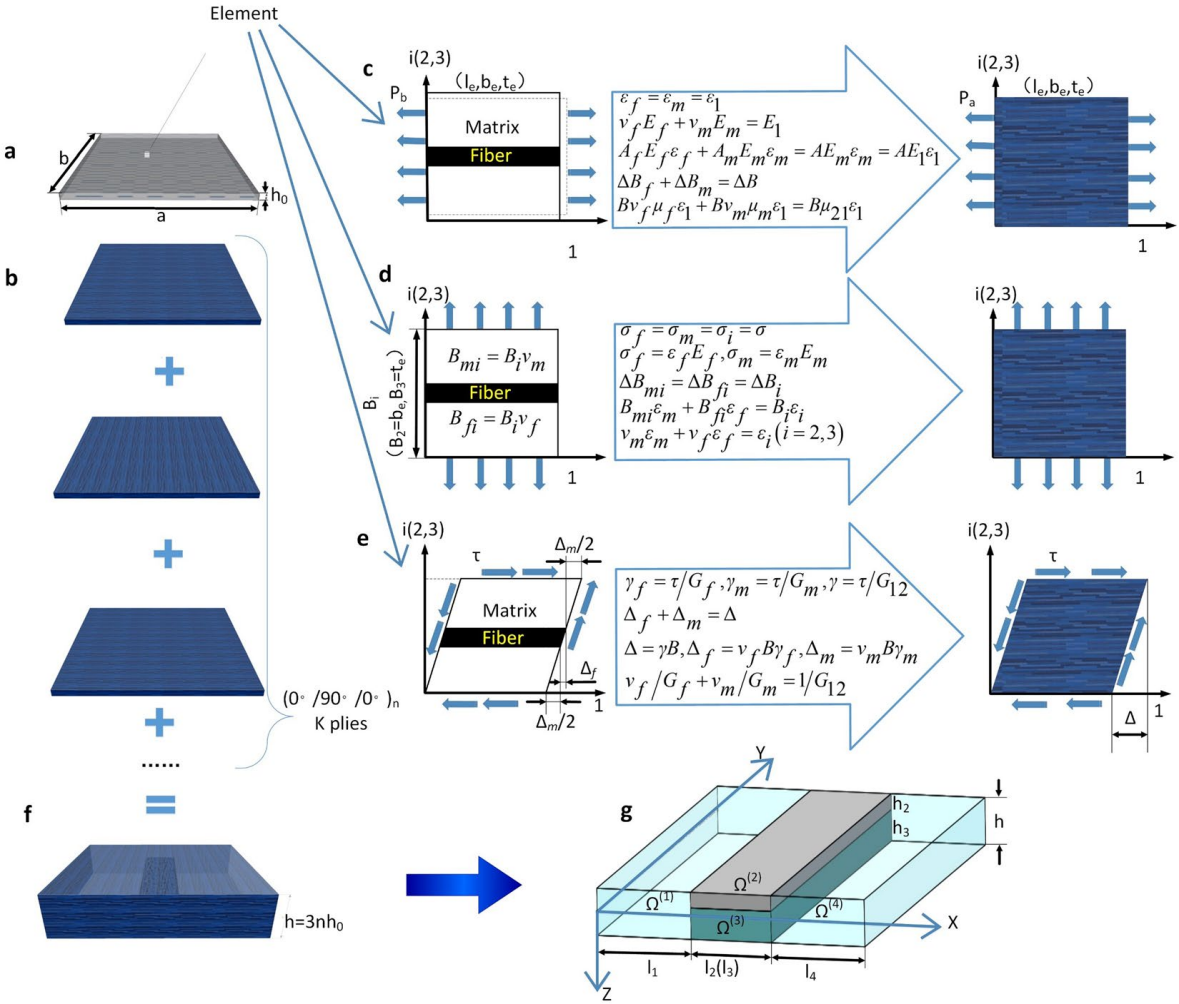
Illustration of material modeling. (**a**) A single layer of unidirectional fiber reinforced composite ply with a continuous matrix and dispersed fibers. (**b**) The equivalent homogeneous, anisotropic composite ply. (**c**) Determination of the equivalent material properties *E*_11_ and *μ*_21_. (**d**) Determination of the equivalent material properties *E*_22_, and *E*_33_. (**e**) Determination of the equivalent in-plane shear modulus *G*_12_. (**f**) Symmetrically cross-plied composite laminate. (**g**) A composite laminate containing a through-width delamination.

Figure 1a–f illustrate the material modeling of a symmetrically cross-plied composite laminate. The laminate consists of unidirectional fiber reinforced composite plies (Fig. 1a). By performing analysis shown in Fig. 1b–e, the FRCP is homogenized as anisotropic plies with the following equivalent material properties of Young’s modulus *E*_11_ and *E*_22_, Poisson’s ratio *μ*_21_ and Shear modulus *G*_12_

1 $$\begin{array}{rcl}{E}_{11} & = & {E}_{m}{v}_{m}+{E}_{f}{v}_{f}\\ {E}_{22} & = & {E}_{33}={E}_{m}{E}_{f}/({E}_{f}{v}_{m}+{E}_{m}{v}_{f})\\ {\mu }_{21} & = & {\mu }_{m}{v}_{m}+{\mu }_{f}{v}_{f}\\ {G}_{12} & = & {G}_{m}{G}_{f}/({G}_{f}{v}_{m}+{G}_{m}{v}_{f})\end{array}$$

where *E*_*m*_, *μ*_*m*_, *G*_*m*_ and *v*_*m*_ are the Young’s modulus, Poisson’s ratio, Shear modulus and volume fraction of the matrix, and *E*_*f*_, *μ*_*f*_, *G*_*f*_ and *v*_*f*_ are the corresponding properties of the fiber. The laminate is manufactured by stacking the fiber reinforced composite plies in an orientation sequence, as shown in Fig. 1f. Let *θ* be the angle of the orientation of *m*-th ply with respect to the horizontal axis in the global coordinate systems, the stiffness coefficient matrix of the *m*-th ply ${({\bar{Q}}_{jk})}_{m}$ in the global coordinate system is given by^35^

2 $${[\begin{array}{c}{\bar{Q}}_{11}\\ {\bar{Q}}_{12}\\ {\bar{Q}}_{22}\\ {\bar{Q}}_{16}\\ {\bar{Q}}_{26}\\ {\bar{Q}}_{66}\end{array}]}_{m}=[\begin{array}{cccc}{c}^{4} & 2{c}^{2}{s}^{2} & {s}^{4} & 4{c}^{2}{s}^{2}\\ {c}^{2}{s}^{2} & {c}^{4}+{s}^{4} & {c}^{2}{s}^{2} & -4{c}^{2}{s}^{2}\\ {s}^{4} & 2{c}^{2}{s}^{2} & {c}^{4} & 4{c}^{2}{s}^{2}\\ {c}^{3}s & c{s}^{3}-{c}^{3}s & -c{s}^{3} & -2cs({c}^{2}-{s}^{2})\\ c{s}^{3} & {c}^{3}s-c{s}^{3} & -{c}^{3}s & 2cs({c}^{2}-{s}^{2})\\ {c}^{2}{s}^{2} & -2{c}^{2}{s}^{2} & {c}^{2}{s}^{2} & {({c}^{2}-{s}^{2})}^{2}\end{array}][\begin{array}{c}{Q}_{11}\\ {Q}_{12}\\ {Q}_{22}\\ {Q}_{66}\end{array}]$$

where *c* = cos*θ*, *s* = sin*θ*, and

3 $$\begin{array}{c}{Q}_{11}=\frac{{E}_{1}}{1-{\mu }_{12}{\mu }_{21}}\quad \quad {Q}_{22}=\frac{{E}_{2}}{1-{\mu }_{12}{\mu }_{21}}\quad {Q}_{66}={G}_{12}\\ {Q}_{12}={\mu }_{12}{Q}_{22}={\mu }_{21}{Q}_{11}\quad {\mu }_{12}{E}_{2}={\mu }_{21}{E}_{1}\end{array}$$

To investigate the influence of the delamination on the dynamic behaviors of the delaminated composite laminates, we consider a rectangular composite lamina of length *a*, width *b*, and height *h*. The composite lamina consists of *K* layers of fiber-reinforced composite ply with a through-width delamination located at depth *h*_2_ from its top surface. The delamination divides the spatial region of the laminate into four sub-laminates of length *l*_*i*_ and height *h*_*i*_, separately referred to as sub-laminate *Ω* ^(*i*)^ (*i* = 1, 2, 3, and 4), as shown in Fig. 1g. The mechanical properties of the membranous stiffness ***A*** ^(*i*)^, the tension-bending coupling stiffness ***B*** ^(*i*)^ and the bending stiffness ***D*** ^(*i*)^ of sub-laminate *Ω* ^(*i*)^ (i = 1, 2, 3, and 4) are given by the following formula^35^

4 $$\begin{array}{rcl}{A}_{jk}^{(i)} & = & \sum _{m=1}^{{K}_{i}}{[{\bar{Q}}_{jk}]}_{m}({h}_{m}-{h}_{m-1}),\\ {B}_{jk}^{(i)} & = & \frac{1}{2}\sum _{m=1}^{{K}_{i}}{[{\bar{Q}}_{jk}]}_{m}({h}_{m}^{2}-{h}_{m-1}^{2}),\\ {D}_{jk}^{(i)} & = & \frac{1}{3}\sum _{m=1}^{{K}_{i}}{[{\bar{Q}}_{jk}]}_{m}({h}_{m}^{3}-{h}_{m-1}^{3}).\end{array}\quad \quad (j,k=1,2,6)$$

where *h*_*m*_ and *h*_*m*−1_ are the location of the *m*-th ply with respect to the neutral axis of sub-laminate *Ω* ^(*i*)^, *K*_*i*_ is the number of plies of *Ω* ^(*i*)^.

In this article, we are interested in analytical investigation on the interaction mechanism between the sub-laminates and its influence on the nonlinear vibration properties. Such analysis requires solving eight nonlinear partial differential equations and thirty-two auxiliary conditions. To clearly present our analytical approach without hindered by tedious calculation, we consider that all the four sub-laminates *Ω* ^(*i*)^ are symmetrically cross-plied. In this way, the in-plane resultant stresses *N* ^*(i)*^(*x*, *y*, *t*) and the bending moment *M* ^*(i)*^(*x*, *y*, *t*) of sub-laminate *Ω* ^(*i*)^ are related to the in-plane strain components *ε* ^*(i)*^(*x*, *y*, *t*) and the change of curvatures *κ* ^*(i)*^(*x*, *y*, *t*), respectively, through the following constitutive relations:

5 $$[{N}^{(i)}(x,y,t)]=[{{\bf{A}}}^{(i)}][{\varepsilon }^{(i)}(x,y,t)],\quad \quad [{M}^{(i)}(x,y,t)]=[{{\bf{D}}}^{(i)}][{\kappa }^{(i)}(x,y,t)].$$

Because of much small thickness dimension comparing to the in-plane dimensions, the transverse displacement at any point in the laminate is represented by the deflection at the corresponding point on the middle plane in the shell theories, i.e., the unit elongation in the transverse direction of the composite laminate *ε*_*z*_ is ignored. For this reason, the material property in the transverse direction of the composite laminate *E*_33_ is not required in shell theories. Such approximation won’t produce obvious error in analyzing the overall responses of the composite laminate. Nevertheless, to scrutinize the nonlinear vibration of the delaminated composite laminate, our discussion is on the conduct of local interaction effects between the sub-laminates.

Prior to the commencement of local deformation, we need to ascertain the material properties of the four sub-laminates in transverse direction. As shown in Fig. 1f, a composite laminate is manufactured by stacking the composite plies in an orientation sequence in which the angle of orientation of each composite ply is inclined in-plane with respect to *x* - or *y* - axis. Therefore, the vertical stacking of the composite plies does not affect the material properties in transverse direction. In other words, the material properties in transverse direction are sensitive to the material properties and the volume frictions of the matrix and the fiber but are insusceptible to the orientation sequence. Following this observation, we draw a very important conclusion that regardless of the stacking sequence and the number of plies, the equivalent Young’s modulus in transverse direction of the four sub-laminates are identical and equal to that of the single ply provided that the composition for each single ply remains unchanged, i.e.

6 $${\bar{E}}_{33}^{(1)}={\bar{E}}_{33}^{(2)}={\bar{E}}_{33}^{(3)}={\bar{E}}_{33}^{(4)}={E}_{33}.$$

### Interaction kinematics of sub-laminates

When the laminate undergoes vibration, it experiences transverse deflection. Once the laminate deviates from its equilibrium position, it starts to bend first at the center of sub-laminate *Ω* ^(2)^. Sub-laminate *Ω* ^(2)^ continues to deflect by pushing sub-laminate *Ω* ^(3)^ to deform downwards simultaneously until both Sub-laminates *Ω* ^(2)^ and *Ω* ^(3)^ reach their equilibrium states. During this process, a reaction force *q* ^*^(*x*, *y*, *t*), called interaction force, is produced between Sub-laminates *Ω* ^(2)^ and *Ω* ^(3)^.

**Figure 2:**
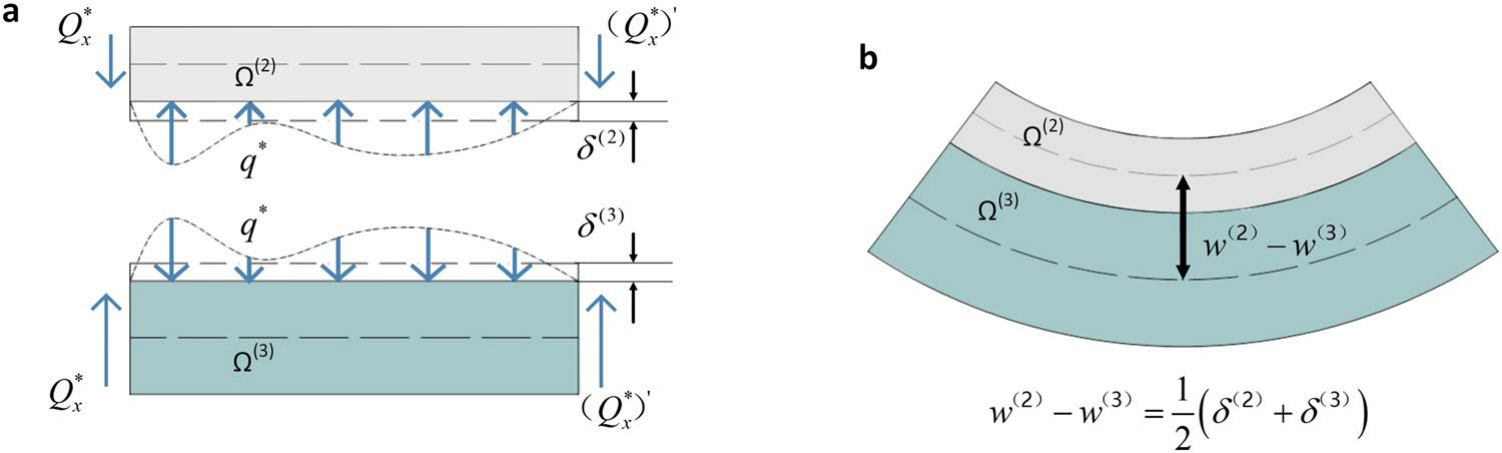
Illustration of the deformation mechanism in one of the contact regions *R*∈[*x*, *x* + *Δx*] between Sub-laminates Ω^(2)^ and Ω^(3)^ during vibration. (**a**) Local interaction kinematics at the interfaces of Sub-laminates Ω^(2)^ and Ω^(3)^. (**b**) A compatiable deformation between macro deflection and micro local deformation of Sub-laminates Ω^(2)^ and Ω^(3)^.

The vibration modes represent the overall response of the delaminated laminate to the external load, thus the amplitudes and the patterns of the external forces, the supporting conditions at the boundary and the geometries of the laminate determine the modes of the vibration. However, the general modes are not the prior to determine the interaction constraint. On the contrary, they depend on the interaction constraint. At any particular moment of the vibration, each of the sub-laminate *Ω* ^(*i*)^ experiences a particular deflection *w* ^(*i*)^(*x*, *y*, *t*) (for i = 1, …, 4). Depending on the amplitudes of *w* ^(2)^ and *w* ^(3)^, contact regions (where *w* ^(2)^ ≥ *w* ^(3)^) and voids (where *w* ^(2)^ < *w* ^(3)^) may form alternately along the interfaces of the delamination between Sub-laminates *Ω* ^(2)^ and *Ω* ^(3)^. Although the contact effects, such as the positions where the contact may occur, the size of the contact areas and the magnitude of the contact force, are still unknown, it does not prevent us from further analyzing. It sounds quite rational to consider that the contact may occur at several regions along the interface of sub-laminates *Ω* ^(2)^ and *Ω* ^(3)^. Denoting the coordinates of one of such regions as *R* ∈ [*x*, *x* + *Δx*], a contact force *q* ^*^(*x*, *y*, *t*) is produced at this place since the deflection of sub-laminates *Ω* ^(2)^ is larger than that of *Ω* ^(3)^, as shown in Fig. 2a. The following analysis is based on Contact Region R, but we will show later in Subsection Nonlinear dynamic stability analysis that the analysis is valid for the entire laminate. As shown in Fig. 2a, the interaction force *q* ^*^(*x*, *y*, *t*) developed at the contact regions produces thickness reductions by amounts of *δ* ^(2)^ and *δ* ^(3)^ for Sub-laminates *Ω* ^(2)^ and *Ω* ^(3)^, respectively

7 $${\delta }^{(2)}(x,y,t)={q}^{\ast }(x,y,t){h}_{2}/{\bar{E}}_{33}^{(2)}$$

8 $${\delta }^{(3)}(x,y,t)={q}^{\ast }(x,y,t){h}_{3}/{\bar{E}}_{33}^{(3)}$$

where *h*_2_ and *h*_3_ are original thickness of *Ω* ^(2)^ and *Ω* ^(3)^, and ${\bar{E}}_{33}^{(2)}$ and ${\bar{E}}_{33}^{(3)}$ are the equivalent Young’s modulus of *Ω* ^(2)^ and *Ω* ^(3)^ in the z-direction. The relative movement of the mid-plane of sub-laminates *Ω* ^(2)^ and *Ω* ^(3)^ is approximately

9 $${w}^{(2)}(x,y,t)-{w}^{(3)}(x,y,t)=\frac{1}{2}[{\delta }^{(2)}(x,y,t)+{\delta }^{(3)}(x,y,t)].$$

Put Eq. (6) into Eqs (7) and (8) and combine with Eq. (9). The kinematic relation between the relative movement *w* ^(2)^–*w* ^(3)^ of Sub-laminates *Ω* ^(2)^ and *Ω* ^(3)^ and their interaction forces *q* ^*^ is found to be

10 $${q}^{\ast }(x,y,t)=k[{w}^{(2)}(x,y,t)-{w}^{(3)}(x,y,t)],$$

where *k* is the interaction factor given by:

11 $$k=2{E}_{33}/h.$$

The interaction brace plays a pivotal role in our problem. It determines both the general response (macro behavior) and the local buckling (micro behavior) of the laminate subjected to the action of the external load.

### Nonlinear theory of dynamic analysis

We consider the composite laminate under the action of an external excitation. The external excitation is resolved into two components: a horizontal one acting in the middle plane of the composite laminate and a transverse one normal to the middle plane. The horizontal component causes the laminate to undergo in-plane or membrane deformation and the transverse component induces out-of-plane deflection or bending in the composite laminate. Therefore, the deformation mechanisms of the composite laminate are analyzed via both membrane and flexural mechanisms of plates. When the in-plane deformations and the flexural deflection of the laminate are small, the membrane and the flexural shell models are analyzed independently. Under such circumstance, we say that the problem is linear. On the contrary, the membrane and the flexural analyses are coupled with each other. We refer to such case as nonlinear problem. In this section, we derive a theory of nonlinear vibration for delaminated composite laminate.

We begin with the nonlinear membrane kinematics. When the delaminated composite laminate is subjected to high levels of acoustic pressure, it undergoes large amplitude vibrations. The large amplitude produces in-plane membrane stretching in the composite laminate which is considered as in-plane force resultants applied to the middle planes of each of the sub-laminate and parallel to their middle planes. In these conditions, the problems are reduced to plane stress problems. Ignoring the body forces and the in-plane inertial forces, the differential equations of equilibrium for membrane deformation of sub-laminate *Ω* ^(*i*)^ are obtained from theory of elasticity as follows:

12 $${N}_{x}^{(i)}{(x,y,t)}_{,x}+{N}_{xy}^{(i)}{(x,y,t)}_{,y}=0,\quad {N}_{xy}^{(i)}{(x,y,t)}_{,x}+{N}_{y}^{(i)}{(x,y,t)}_{,y}=0,\quad {\rm{for}}\,i=1,\ldots ,4$$

where a comma denotes partial differentiation with respect to the corresponding coordinates. Equation (12) are satisfied by introducing a stress function *ψ* ^(*i*)^ (*x*, *y*, *t*) such that

13 $${N}_{x}^{(i)}={\psi }_{,yy}^{(i)},\quad {N}_{y}^{(i)}={\psi }_{,xx}^{(i)},\quad {N}_{xy}^{(i)}=-{\psi }_{,xy}^{(i)}.$$

Define the in-plane displacements in the middle plane of sub-laminate *Ω* ^(*i*)^ as *u* ^(*i*)^(*x*, *y*, *t*) and *v* ^(*i*)^(*x*, *y*, *t*). The in-plane strain components *ε* ^(*i*)^(*x*, *y*, *t*) are related to the in-plane displacements and the transverse deflection through von Karman nonlinear geometric kinematics as follows^36, 37^:

14 $${\varepsilon }_{x}^{(i)}={u}_{,x}^{(i)}+\frac{1}{2}{({w}_{,x}^{(i)})}^{2},\quad {\varepsilon }_{y}^{(i)}={v}_{,y}^{(i)}+\frac{1}{2}{({w}_{,y}^{(i)})}^{2},\quad {\gamma }_{xy}^{(i)}={u}_{,y}^{(i)}+{v}_{,x}^{(i)}+{w}_{,x}^{(i)}\cdot {w}_{,y}^{(i)}.$$

Eliminating *u* ^(*i*)^ and *v* ^(*i*)^ from Eq. (14) and using Eqs (5) and (13) we have

15 $${A}_{11}^{{(i)}^{\ast }}{\psi }_{,yyyy}^{(i)}+2({A}_{12}^{{(i)}^{\ast }}+\frac{1}{2}{A}_{66}^{{(i)}^{\ast }}){\psi }_{,xxyy}^{(i)}+{A}_{22}^{{(i)}^{\ast }}{\psi }_{,xxxx}^{(i)}={({w}_{,xy}^{(i)})}^{2}-{w}_{,xx}^{(i)}\cdot {w}_{,yy}^{(i)},\quad \quad {\rm{for}}\,i=1,\ldots ,4.$$

where ***A*** ^(*i*)*^ is the inverse of the membranous stiffness ***A*** ^(*i*)^. Equation (15) is the compatibility equation for in-plane membrane deformation of Sub- laminate *Ω* ^(*i*)^ in terms of the stress function *ψ* ^(*i*)^ and the transverse deflection *w* ^(*i*)^.

Next, we examine the nonlinear flexural mechanism of the composite laminate. From the point view of shell theory, the nonlinear problem differs from the linear problem mainly on the treatment of the membrane forces. In linear problem, the membrane forces in the middle plane of the laminate are fixed, while in nonlinear problem they change with the increasing of the amplitude of the deflection and the influence of the membrane forces on the force equilibrium in transverse direction cannot be ignored. For this reason, the differential equation of equilibrium in transverse direction and the moment equilibrium for nonlinear bending of sub-laminate *Ω* ^(*i*)^ take the following forms^37^:

16 $$\begin{array}{rcl}{Q}_{x,x}^{(i)}+{Q}_{x,x}^{(i)}-{N}_{x}^{(i)}\cdot {\kappa }_{x}^{(i)}+2{N}_{xy}^{(i)}\cdot {\kappa }_{xy}^{(i)}-{N}_{y}^{(i)}\cdot {\kappa }_{y}^{(i)}+{m}^{(i)}{w}_{,tt}^{(i)} & = & {q}^{(i)},\\ {M}_{x,x}^{(i)}+{M}_{xy,y}^{(i)} & = & {Q}_{x}^{(i)},\\ {M}_{xy,x}^{(i)}+{M}_{y,y}^{(i)} & = & {Q}_{y}^{(i)}.\end{array}$$

where *Q*_*j*_^(*i*)^(*x*, *y*, *t*) is the transverse shear forces of sub-laminate *Ω* ^(*i*)^, *m* ^(*i*)^ is the mass moment of inertia, *q* ^(*i*)^(*x*, *y*, *t*) is the distributed load in transverse direction applied to Sub-laminates *Ω* ^(*i*)^, and the changes of curvatures *κ* ^*(i)*^(*x*, *y*, *t*) are given by

17 $${\kappa }_{x}^{(i)}=-{w}_{,xx}^{(i)},\quad {\kappa }_{y}^{(i)}=-{w}_{,yy}^{(i)},\quad {\kappa }_{xy}^{(i)}=-{w}_{,xy}^{(i)}.$$

Substituting the last two of Eq. (16) into the first equation and introducing Eqs (5), (13) and (17) we have

18 $${D}_{11}^{(i)}{w}_{,xxxx}^{(i)}+2({D}_{12}^{(i)}+{D}_{66}^{(i)}){w}_{,xxyy}^{(i)}+{D}_{22}^{(i)}{w}_{,yyyy}^{(i)}-{\psi }_{,yy}^{(i)}\cdot {w}_{,xx}^{(i)}+2{\psi }_{,xy}^{(i)}\cdot {w}_{,xy}^{(i)}-{\psi }_{,xx}^{(i)}\cdot {w}_{,yy}^{(i)}+{m}^{(i)}{w}_{,tt}^{(i)}={q}^{(i)}.$$

In Eq. (18), the term *m* ^(*i*)^ *w*_,*tt*_^(*i*)^ represents the component of inertial force along with nonuniform motion. Thus, Eq. (18) is also known as the equation of motion for nonlinear vibration of Sub-laminates *Ω* ^(*i*)^.

Now, we need to consider the influence of the interaction penalty between sub-laminates. Due to the interaction forces *q* ^*^(*x*, *y*, *t*) between sub-laminates, the distributed load acting on each sub-laminate *q* ^(*i*)^(*x*, *y*, *t*) is not the same. The interaction forces transmit the external force from sub-laminate *Ω* ^(2)^ to *Ω* ^(3)^, causing a reallocation of the external force *q*(*x*, *y*, *t*) between sub-laminates *Ω* ^(2)^ and *Ω* ^(3)^: that is a net force of *q*(*x*, *y*, *t*) *- q* ^*^(*x*, *y*, *t*) on sub-laminates *Ω* ^(2)^ and *q* ^*^(*x*, *y*, *t*) on *Ω* ^(3)^. With the available interaction forces *q* ^*^(*x*, *y*, *t*) given in Eqs (10) and (11) the distributed load on each sub-laminate *q* ^(*i*)^ is reapportioned as follows:

19 $${q}^{(i)}=\{\begin{array}{ll}q & for\quad Sub-la\,{\rm{\min }}\,ate\quad {{\rm{\Omega }}}^{(1)}\quad and\quad {{\rm{\Omega }}}^{(4)}\\ q-{q}^{\ast }=q-k({w}^{(2)}-{w}^{(3)}) & for\quad Sub-la\,{\rm{\min }}\,ate\quad {{\rm{\Omega }}}^{(2)}\\ {q}^{\ast }=k({w}^{(2)}-{w}^{(3)}) & for\quad Sub-la\,{\rm{\min }}\,ate\quad {{\rm{\Omega }}}^{(3)}\end{array}$$

Replacing Eq. (19) into Eq. (18) and combining Eq. (15), we derive the governing equations for the nonlinear vibration of Sub-laminates *Ω* ^(*i*)^.

### Delamination integration

Although we have established the governing equations of nonlinear vibration for each particular sub-laminate *Ω* ^(*i*)^, the four sub-laminates are still independent with each other. Except the interaction effect between *Ω* ^(*2*)^ and *Ω* ^(*3*)^, we need to impose constrains to make the four sub-laminates be the four parts of a single delaminated composite laminate. Therefore, we enforce the equilibrium conditions and compatibility conditions between the sub-laminates to unite them as a monolithic entity with a delamination integrated.

The equilibrium conditions require that the moments, the shear forces, the in-plane forces must be balanced at the fronts of delamination. The continuity conditions mean that the transverse deflection, the slope, and the in-plane displacements must be compatible. They are

At *x* = *l*_1_

20 $$\begin{array}{llll}{N}_{x}^{(1)}={N}_{x}^{(2)}+{N}_{x}^{(3)} & {N}_{xy}^{(1)}={N}_{xy}^{(2)}+{N}_{xy}^{(3)} & {M}_{x}^{(1)}={M}_{x}^{(2)}+{M}_{x}^{(3)} & {Q}_{x}^{(1)}={Q}_{x}^{(2)}+{Q}_{x}^{(3)}\\ {w}^{(1)}={w}^{(2)} & {w}_{,x}^{(1)}={w}_{,x}^{(2)} & {w}^{(1)}={w}^{(3)} & {w}_{,x}^{(1)}={w}_{,x}^{(3)}\\ {u}^{(1)}={u}^{(2)} & {u}^{(1)}={u}^{(3)} & {v}^{(1)}={v}^{(2)} & {v}^{(1)}={v}^{(3)}\end{array}$$

At *x* = *l*_1_ + *l*_2_

21 $$\begin{array}{llll}{N}_{x}^{(4)}={N}_{x}^{(2)}+{N}_{x}^{(3)} & {N}_{xy}^{(4)}={N}_{xy}^{(2)}+{N}_{xy}^{(3)} & {M}_{x}^{(4)}={M}_{x}^{(2)}+{M}_{x}^{(3)} & {Q}_{x}^{(4)}={Q}_{x}^{(2)}+{Q}_{x}^{(3)}\\ {w}^{(4)}={w}^{(2)} & {w}_{,x}^{(4)}={w}_{,x}^{(2)} & {w}^{(4)}={w}^{(3)} & {w}_{,x}^{(4)}={w}_{,x}^{(3)}\\ {u}^{(4)}={u}^{(2)} & {u}^{(4)}={u}^{(3)} & {v}^{(4)}={v}^{(2)} & {v}^{(4)}={v}^{(3)}\end{array}$$

In Eqs (20) and (21) the moment *M*_*x*_^(*i*)^(*x*, *y*, *t*), the shear force *Q*_*x*_^(*i*)^(*x*, *y*, *t*), the in-plane displacements *u* ^(*i*)^(*x*, *y*, *t*) and *v* ^(*i*)^(*x*, *y*, *t*) are related to the transverse deflection *w* ^(*i*)^ and the stress function *ψ* ^(*i*)^ through the following equations, respectively,

22 $$\begin{array}{ll}{M}_{x}^{(i)}={D}_{11}^{(i)}{w}_{,xx}^{(i)}+{D}_{12}^{(i)}{w}_{,yy}^{(i)}, & {Q}_{x}^{(i)}={D}_{11}^{(i)}{w}_{,xxx}^{(i)}+({D}_{12}^{(i)}+2{D}_{66}^{(i)}){w}_{,xyy}^{(i)},\\ {u}^{(i)}=\int [{A}_{11}^{\ast (i)}{\psi }_{,yy}^{(i)}+{A}_{12}^{\ast (i)}{\psi }_{,xx}^{(i)}-\frac{1}{2}{({w}_{,x}^{(i)})}^{2}]dx, & {v}^{(i)}=\int [{A}_{12}^{\ast (i)}{\psi }_{,yy}^{(i)}+{A}_{22}^{\ast (i)}{\psi }_{,xx}^{(i)}-\frac{1}{2}{({w}_{,y}^{(i)})}^{2}]dy.\end{array}$$

Equations (15), (18–22) constitute the general theory for nonlinear vibration of fiber-reinforced composite laminates containing an embedded delamination. The theory accounts for large geometric deformation in transverse direction, the in-plane membrane stretching, and the coupling interaction penalty at the interfaces of the delamination. It is highly nonlinear—not only because of the coupling of membrane mechanism and flexural mechanism within each sub-laminate, but also due to the coupling of the transverse deflection between sub-laminates.

### Nonlinear dynamic stability analysis

As an application of the proposed theory, we analyze the free vibration of a delaminated composite laminate simply supported at its four edges. The laminate is made of graphite/epoxy composite plies with equivalent material properties of the longitudinal modulus *E*_11_ = 140 GPa, the transverse modulus *E*_22_ = 10 GPa, the shear modulus *G*_12_ = 5 GPa, and the Poisson’s ratios *μ*_12_ = 0.3. It contains a though-width delamination symmetrically located at its center and is referred to a global coordinate system (*x*, *y*, *z*) centered at the left corner of the middle surface of the sublaminate, in which *x* and *y* are in the longitudinal and breadth directions and *z* is in the direction of the inward normal to the middle surface.

In a real problem, mechanical behaviors depend on the dimensions of the physical quantities. To establish a general model with performances insusceptible to the dimensions of the physical quantities, we introduce the following non-dimensional parameters:

23 $$\begin{array}{c}\overline{x}=\frac{x}{a},\overline{y}=\frac{y}{b},\overline{k}=k\cdot \frac{{h}_{0}^{2}}{{A}_{22}^{(1)}},\overline{{A}_{ij}^{\ast (i)}}={A}_{ij}^{\ast (i)}{A}_{22}^{(1)},\overline{\omega }=\sqrt{\frac{m{h}_{0}^{2}}{{A}_{22}^{(1)}}}\omega ,\overline{{M}_{x}^{(i)}}=\frac{{M}_{x}^{(i)}}{{A}_{22}^{(1)}{h}_{0}},\\ \overline{{D}_{ij}^{(i)}}=\frac{{D}_{ij}^{(i)}}{{A}_{22}^{(1)}{h}_{0}^{2}},{\alpha }_{i}=\frac{{h}_{0}}{{l}_{i}},\beta =\frac{{h}_{0}}{b},{\tau }_{i}=\frac{{h}_{i}}{h},{\overline{w}}^{(i)}=\frac{{w}^{(i)}}{{h}_{0}},\overline{{Q}_{x}^{(i)}}=\frac{{Q}_{x}^{(i)}}{{A}_{22}^{(1)}},\overline{t}=t\cdot \omega \end{array}$$

where *ω* is the frequency of free vibration of the composite lamina. The free vibration of the composite laminate is an eigenvalue problem, thus the frequency of the free vibration *ω* is a characteristic value of the composite laminate and unaffected by external forces, such as the transverse load *q* on the surfaces or in-plane forces *N*_*ij*_ at the edges. Under this circumstance, the membrane forces induced by the transverse deflection do not need to be considered, i.e. Equation (15) is not required^38^. Substituting Eq. (23) into (18) and setting *ψ*_,*ij*_^(*i*)^ and *q* to be zero, the governing equation for free vibration of the delaminated composite laminate is rewritten from Eqs (18) and (19) as the following using Eq. (23):

24 $${\alpha }_{i}^{4}\overline{{D}_{11}^{(i)}}{\overline{w}}_{,\bar{x}\bar{x}\bar{x}\bar{x}}^{(i)}+2{\alpha }_{i}^{2}{\beta }^{2}(\overline{{D}_{12}^{(i)}}+\overline{{D}_{66}^{(i)}}){\overline{w}}_{,\bar{x}\bar{x}\bar{y}\bar{y}}^{(i)}+{\beta }^{4}\overline{{D}_{22}^{(3)}}{\overline{w}}_{,\bar{y}\bar{y}\bar{y}\bar{y}}^{(i)}+\overline{{\tau }_{i}}\overline{\omega }{\overline{w}}_{,\bar{t}\bar{t}}^{(i)}=0\quad i=1{\&}4\quad for\quad {{\rm{\Omega }}}^{(1)}{\&}{{\rm{\Omega }}}^{(4)}$$

25 $${\alpha }_{2}^{4}\overline{{D}_{11}^{(2)}}{\overline{w}}_{,\bar{x}\bar{x}\bar{x}\bar{x}}^{(2)}+2{\alpha }_{2}^{2}{\beta }^{2}(\overline{{D}_{12}^{(2)}}+\overline{{D}_{66}^{(2)}}){\overline{w}}_{,\bar{x}\bar{x}\bar{y}\bar{y}}^{(2)}+{\beta }^{4}\overline{{D}_{22}^{(2)}}{\overline{w}}_{,\bar{y}\bar{y}\bar{y}\bar{y}}^{(2)}+\overline{k}({\overline{w}}_{2}-{\overline{w}}_{3})+\overline{{\tau }_{2}}\overline{\omega }{\overline{w}}_{,\bar{t}\bar{t}}^{(2)}=0$$

26 $${\alpha }_{3}^{4}\overline{{D}_{11}^{(3)}}{\overline{w}}_{,\bar{x}\bar{x}\bar{x}\bar{x}}^{(3)}+2{\alpha }_{3}^{2}{\beta }^{2}(\overline{{D}_{12}^{(3)}}+\overline{{D}_{66}^{(3)}}){\overline{w}}_{,\bar{x}\bar{x}\bar{y}\bar{y}}^{(3)}+{\beta }^{4}\overline{{D}_{22}^{(3)}}{\overline{w}}_{,\bar{y}\bar{y}\bar{y}\bar{y}}^{(3)}-\overline{k}({\overline{w}}_{2}-{\overline{w}}_{3})+\overline{{\tau }_{3}}\overline{\omega }{\overline{w}}_{,\bar{t}\bar{t}}^{(3)}=0$$

As the membrane forces and deformations are not included for the problem discussed herein, we need to remove these terms from equilibrium and continuity conditions in Eqs (20) and (21). The remaining formulas are the equilibrium of bending moment *M*_*x*_ and shear force *Q*_*x*_ and the continuity of the deflection *w* and it’s slope *w*_,*x*_, which are expressed in nondimensional forms as follows:

27 $$\begin{array}{c}\overline{{D}_{11}^{(1)}}{\alpha }_{1}^{2}{\overline{w}}^{(1)}{(1,\overline{y},\overline{t})}_{,\bar{x}\bar{x}}+\overline{{D}_{12}^{(1)}}{\beta }^{2}{\overline{w}}^{(1)}{(1,\overline{y},\overline{t})}_{,\bar{y}\bar{y}}\\ \quad \quad \quad \quad =\overline{{D}_{11}^{(2)}}{\alpha }_{2}^{2}{\overline{w}}^{(2)}{(0,\overline{y},\overline{t})}_{,\bar{x}\bar{x}}+\overline{{D}_{11}^{(2)}}{\beta }^{2}{\overline{w}}^{(2)}{(0,\overline{y},\overline{t})}_{,\bar{y}\bar{y}}+\overline{{D}_{11}^{(3)}}{\alpha }_{3}^{2}{\overline{w}}^{(3)}{(0,\overline{y},\overline{t})}_{,\bar{x}\bar{x}}+\overline{{D}_{11}^{(3)}}{\beta }^{2}{\overline{w}}^{(3)}{(0,\overline{y},\overline{t})}_{,\bar{y}\bar{y}}\\ \overline{{D}_{11}^{(4)}}{\alpha }_{4}^{2}{\overline{w}}^{(4)}{(0,\overline{y},\overline{t})}_{,\bar{x}\bar{x}}+\overline{{D}_{12}^{(4)}}{\beta }^{2}{\overline{w}}^{(4)}{(0,\overline{y},\overline{t})}_{,\bar{y}\bar{y}}\\ \quad \quad \quad \quad =\overline{{D}_{11}^{(2)}}{\alpha }_{2}^{2}{\overline{w}}^{(2)}{(1,\overline{y},\overline{t})}_{,\bar{x}\bar{x}}+\overline{{D}_{11}^{(2)}}{\beta }^{2}{\overline{w}}^{(2)}{(1,\overline{y},\overline{t})}_{,\bar{y}\bar{y}}+\overline{{D}_{11}^{(3)}}{\alpha }_{3}^{2}{\overline{w}}^{(3)}{(1,\overline{y},\overline{t})}_{,\bar{x}\bar{x}}+\overline{{D}_{11}^{(3)}}{\beta }^{2}{\overline{w}}^{(3)}{(1,\overline{y},\overline{t})}_{,\bar{y}\bar{y}}\end{array}$$

28 $$\begin{array}{c}\overline{{D}_{11}^{(1)}}{\alpha }_{1}^{3}{\overline{w}}^{(1)}{(1,\overline{y},\overline{t})}_{,\bar{x}\bar{x}\bar{x}}\\ \quad +(\overline{{D}_{12}^{(1)}}+2\overline{{D}_{66}^{(1)}}){\alpha }_{1}{\beta }^{2}{\overline{w}}^{(1)}{(1,\overline{y},\overline{t})}_{,\bar{x}\bar{y}\bar{y}}=\overline{{D}_{11}^{(2)}}{\alpha }_{2}^{3}{\overline{w}}^{(2)}{(0,\overline{y},\overline{t})}_{,\bar{x}\bar{x}\bar{x}}\\ \quad \quad \quad \quad \quad \quad \quad \quad \quad \quad \quad \quad \quad \quad \quad \quad \quad \quad +(\overline{{D}_{12}^{(2)}}+2\overline{{D}_{66}^{(2)}}){\alpha }_{2}{\beta }^{2}{\overline{w}}^{(2)}{(0,\overline{y},\overline{t})}_{,\bar{x}\bar{y}\bar{y}}\\ \quad \quad \quad \quad \quad \quad \quad \quad \quad \quad \quad \quad \quad \quad \quad \quad \quad \quad +\overline{{D}_{11}^{(3)}}{\alpha }_{3}^{3}{\overline{w}}^{(3)}{(0,\overline{y},\overline{t})}_{,\bar{x}\bar{x}\bar{x}}(\overline{{D}_{12}^{(3)}}+2\overline{{D}_{66}^{(3)}})\\ \quad \quad \quad \quad \quad \quad \quad \quad \quad \quad \quad \quad \quad \quad \quad \quad \quad \quad \times {\alpha }_{3}{\beta }^{2}{\overline{w}}^{(3)}{(0,\overline{y},\overline{t})}_{,\bar{x}\bar{y}\bar{y}}\\ \overline{{D}_{11}^{(4)}}{\alpha }_{4}^{3}{\overline{w}}^{(4)}{(0,\overline{y},\overline{t})}_{,\bar{x}\bar{x}\bar{x}}\\ \quad +(\overline{{D}_{12}^{(4)}}+2\overline{{D}_{66}^{(4)}}){\alpha }_{4}{\beta }^{2}{\overline{w}}^{(4)}{(0,\overline{y},\overline{t})}_{,\bar{x}\bar{y}\bar{y}}=\overline{{D}_{11}^{(2)}}{\alpha }_{2}^{3}{\overline{w}}^{(2)}{(1,\overline{y},\overline{t})}_{,\bar{x}\bar{x}\bar{x}}+(\overline{{D}_{12}^{(2)}}+2\overline{{D}_{66}^{(2)}})\\ \quad \quad \quad \quad \quad \quad \quad \quad \quad \quad \quad \quad \quad \quad \quad \quad \quad \quad \times {\alpha }_{2}{\beta }^{2}{\overline{w}}^{(2)}{(1,\overline{y},\overline{t})}_{,\bar{x}\bar{y}\bar{y}}\\ \quad \quad \quad \quad \quad \quad \quad \quad \quad \quad \quad \quad \quad \quad \quad \quad \quad \quad +\overline{{D}_{11}^{(3)}}{\alpha }_{3}^{3}{\overline{w}}^{(3)}{(1,\overline{y},\overline{t})}_{,\bar{x}\bar{x}\bar{x}}+(\overline{{D}_{12}^{(3)}}+2\overline{{D}_{66}^{(3)}})\\ \quad \quad \quad \quad \quad \quad \quad \quad \quad \quad \quad \quad \quad \quad \quad \quad \quad \quad \times {\alpha }_{3}{\beta }^{2}{\overline{w}}^{(3)}{(1,\overline{y},\overline{t})}_{,\bar{x}\bar{y}\bar{y}}\end{array}$$

29 $$\begin{array}{rcl}{\overline{w}}^{(1)}(1,\overline{y},\overline{t}) & = & {\overline{w}}^{(2)}(0,\overline{y},\overline{t})\\ {\overline{w}}^{(1)}(1,\overline{y}) & = & {\overline{w}}^{(3)}(0,\overline{y},\overline{t})\\ {\alpha }_{1}{\overline{w}}^{(1)}{(1,\overline{y},\overline{t})}_{,\bar{x}} & = & {\alpha }_{2}{\overline{w}}^{(2)}{(0,\overline{y},\overline{t})}_{,\bar{x}}\\ {\alpha }_{1}{\overline{w}}^{(1)}{(1,\overline{y},\overline{t})}_{,\bar{x}} & = & {\alpha }_{3}{\overline{w}}^{(3)}{(0,\overline{y},\overline{t})}_{,\bar{x}}\end{array}\quad \quad \begin{array}{rcl}{\overline{w}}^{(4)}(0,\overline{y},\overline{t}) & = & {\overline{w}}^{(2)}(1,\overline{y},\overline{t})\\ {\overline{w}}^{(4)}(0,\overline{y},\overline{t}) & = & {\overline{w}}^{(3)}(1,\overline{y},\overline{t})\\ {\alpha }_{4}{\overline{w}}^{(4)}{(0,\overline{y},\overline{t})}_{,\bar{x}} & = & {\alpha }_{2}{\overline{w}}^{(2)}{(1,\overline{y},\overline{t})}_{,\bar{x}}\\ {\alpha }_{4}{\overline{w}}^{(4)}{(0,\overline{y},\overline{t})}_{,\bar{x}} & = & {\alpha }_{3}{\overline{w}}^{(3)}{(1,\overline{y},\overline{t})}_{,\bar{x}}\end{array}$$

The simply supported laminate cannot support the moments at its four edges and is restricted to move transversely at these places. Thus the boundary conditions in nondimensional forms for this problem are

30 $$\begin{array}{rcl}{\overline{w}}^{(1)}(0,\overline{y},\overline{t}) & = & 0\quad \quad \overline{{D}_{11}^{(1)}}{\alpha }_{1}^{2}{\overline{w}}^{(1)}{(0,\overline{y},\overline{t})}_{,\bar{x}\bar{x}}+\overline{{D}_{12}^{(1)}}{\beta }^{2}{\overline{w}}^{(1)}{(0,\overline{y},\overline{t})}_{,\bar{y}\bar{y}}=0\\ {\overline{w}}^{(4)}(1,\overline{y},\overline{t}) & = & 0\quad \quad \overline{{D}_{11}^{(4)}}{\alpha }_{4}^{2}{\overline{w}}^{(4)}{(1,\overline{y},\overline{t})}_{,\bar{x}\bar{x}}+\overline{{D}_{12}^{(4)}}{\beta }^{2}{\overline{w}}^{(4)}{(1,\overline{y},\overline{t})}_{,\bar{y}\bar{y}}=0\end{array}$$

31 $$\begin{array}{rcl}{\overline{w}}^{(i)}(\overline{x},0,\overline{t}) & = & 0\quad \quad \overline{{D}_{12}^{(i)}}{\alpha }_{i}^{2}{\overline{w}}^{(i)}{(\overline{x},0,\overline{t})}_{,\bar{x}\bar{x}}+\overline{{D}_{22}^{(i)}}{{\rm{\beta }}}^{2}{\overline{w}}^{(i)}{(\overline{x},0,\overline{t})}_{,\bar{y}\bar{y}}=0\\ {\overline{w}}^{(i)}(\overline{x},1,\overline{t}) & = & 0\quad \quad \overline{{D}_{12}^{(i)}}{\alpha }_{i}^{2}{\overline{w}}^{(i)}{(\overline{x},1,\overline{t})}_{,\bar{x}\bar{x}}+\overline{{D}_{22}^{(i)}}{{\rm{\beta }}}^{2}{\overline{w}}^{(i)}{(\overline{x},1,\overline{t})}_{,\bar{y}\bar{y}}=0\end{array}\quad ({\rm{for}}\,{\rm{i}}=1,2,3\,{\rm{and}}\,4)$$

We now need to solve the dimensionless formulas of Eqs (24–26) by satisfying auxiliary conditions Eqs (27–31) to obtain the free vibration modes of the delaminated composite laminate. The process has been accomplished and is shown in section Method for a better description. Here we write down the solution in order to perform further analysis

32 $$\begin{array}{rcl}{\bar{w}}^{(i)}(\bar{x},\bar{y},\bar{t}) & = & ({A}^{(i)}\,\cos \,\bar{t}+{B}^{(i)}\,\sin \,\bar{t})[{G}_{1}^{(i)}{e}^{{\lambda }_{G1}^{(i)}\bar{x}}+{G}_{2}^{(i)}{e}^{-{\lambda }_{G1}^{(i)}\bar{x}}+{G}_{3}^{(i)}\,\cos ({\lambda }_{G2}^{(i)}\bar{x})\\  &  & +{G}_{4}^{(i)}\,\sin ({\lambda }_{G2}^{(i)}\bar{x})]\sin (\pi \bar{y})\quad for\quad {{\rm{\Omega }}}^{(1)}{\&}{{\rm{\Omega }}}^{(4)}\end{array}$$

33 $$\begin{array}{c}{\bar{w}}^{(i)}(\bar{x},\bar{y},\bar{t})=({A}^{(i)}\,\cos \,\bar{t}+{B}^{(i)}\,\sin \,\bar{t})\{[{G}_{1}^{(i)}{e}^{{\lambda }_{G1}^{(i)}\bar{x}}+{G}_{2}^{(i)}{e}^{-{\lambda }_{G1}^{(i)}\bar{x}}\\ \quad \quad \quad \quad \quad \,\,\,+{G}_{3}^{(i)}\,\cos ({\lambda }_{G2}^{(i)}\bar{x})+{G}_{4}^{(i)}\,\sin ({\lambda }_{G2}^{(i)}\bar{x})]\\ \quad \quad \quad \quad \quad \,\,\,+[{L}_{1}^{(i)}{e}^{{\lambda }_{L}\bar{x}}+{L}_{2}^{(i)}{e}^{-{\lambda }_{L}\bar{x}}+{L}_{3}^{(i)}\,\cos ({\lambda }_{L}\bar{x})\\ \quad \quad \quad \quad \quad \,\,\,+{L}_{4}^{(i)}\,\sin ({\lambda }_{L}\bar{x})]\}\,\sin (\pi \bar{y})\quad for\quad {\Omega }^{(2)}{\&}{{\rm{\Omega }}}^{(3)}\end{array}$$

where *G*_j_^(*i*)^ and *L*_*j*_^(*i*)^ are the coefficient constants of the global vibration mode and local buckling mode, *λ*_*G*_^(*i*)^ and *λ*_*L*_ are the modal parameter of global vibration and local buckling. The modal parameters *λ*_*G*_^(*i*)^ and *λ*_*L*_ are found from the governing equation as follows (See section Method for detail):

34 $$\begin{array}{rcl}{\lambda }_{G1}^{(i)} & = & \mathrm{Re}[{b}_{i}/(2{a}_{i})\pm \sqrt{({{b}_{i}}^{2}-4{a}_{i}{c}_{i})/{(2{a}_{i})}^{2}}{]}^{1/2},\\ {\lambda }_{G2}^{(i)} & = & \mathrm{Im}[{b}_{i}/(2{a}_{i})\pm \sqrt{({{b}_{i}}^{2}-4{a}_{i}{c}_{i})/{(2{a}_{i})}^{2}}{]}^{1/2},\quad {\rm{for}}\,i=1{\&}4\,({{\rm{\Omega }}}^{(1)}{\&}{{\rm{\Omega }}}^{(4)})\end{array}$$

35 $$\begin{array}{c}{\lambda }_{G1}^{(2)}={\lambda }_{G1}^{(3)}={\rm{Re}}{[\frac{({b}_{2}+{b}_{3})\pm \sqrt{{({b}_{2}+{b}_{3})}^{2}-4({a}_{2}+{a}_{3})({c}_{2}+{c}_{3})}}{2({a}_{2}+{a}_{3})}]}^{1/2},\\ {\lambda }_{G2}^{(2)}={\lambda }_{G2}^{(3)}={\rm{Im}}{[\frac{({b}_{2}+{b}_{3})\pm \sqrt{{({b}_{2}+{b}_{3})}^{2}-4({a}_{2}+{a}_{3})({c}_{2}+{c}_{3})}}{2({a}_{2}+{a}_{3})}]}^{1/2},\\ {\lambda }_{L}={|\frac{({a}_{2}+{a}_{3})\bar{k}}{{a}_{2}{a}_{3}}|}^{\frac{1}{4}},\,\,{\rm{for}}\,{\Omega }^{(2)}\,{\&}\,{\Omega }^{(3)}\end{array}$$

where *a*_*i*_, *b*_*i*_ and *c*_*i*_ are constants determined by the geometric parameters and material properties of Sublaminate *Ω* ^(*i*)^ as follows:

36 $${a}_{i}=\overline{{D}_{11}^{(i)}}{\alpha }_{i}^{4}\quad {b}_{i}=2(\overline{{D}_{12}^{(i)}}+2\overline{{D}_{66}^{(i)}}){\alpha }_{i}^{2}{\beta }^{2}{(n\pi )}^{2}\quad {c}_{i}=\overline{{D}_{22}^{(i)}}{\beta }^{4}{(n\pi )}^{4}-{\bar{\tau }}_{i}{\bar{\omega }}^{2},\quad \quad ({\rm{for}}\,i=1\ldots 4)$$

The coefficient constants for Sub-laminates *Ω* ^(*2*)^ and *Ω* ^(*3*)^ are also found to be related to each other as follows:

37 $${G}_{j}^{(3)}\approx {G}_{j}^{(2)},\quad \quad {L}_{j}^{(3)}=-\iota {L}_{j}^{(2)},\quad \quad ({\rm{for}}\,j=1\ldots 4)$$

where

38 $$\iota =({a}_{2}{({\lambda }_{L})}^{4}-{b}_{2}{({\lambda }_{L})}^{2}+({c}_{2}-\bar{k}))/\bar{k}.$$

Equations (32–38) are the solutions of the vibration mode. We have mentioned in sub section of Interaction kinematics of sub-laminates that the solutions are valid for Region *R*. However Region *R* is just arbitrarily chosen. It denotes any one of the contact regions. If we use different contact area to analysis, we obtain the same solutions given in (32–38). It means that Eqs (32–38) are valid for all the contact regions. We know that the contact regions and the voids are sequentially distributed along the interfaces of the delamination. Then when we plot out Eqs (32) and (33) along the entire length of the laminate, we can identify the contact regions where *w* ^(2)^ > *w* ^(3)^ and the voids where *w* ^(2)^ < *w* ^(3)^.

Equations (32) and (33) manifests that the nonlinear free vibration of the delaminated composite laminate consists of global vibration and local buckling. In particular, each of the four sublaminate undergoes a global vibration, but the local buckling only occurs between *Ω* ^(*2*)^ and *Ω* ^(*3*)^. In the rest of this section we will explain how the performances of the global mode are different from those of the local buckling.

Going back to Eqs (1–4), we note that Sub-laminates *Ω* ^(*1*)^ and *Ω* ^(*4*)^ possess the same material properties, that is **A** ^(1)^ = **A** ^(4)^, **D** ^(1)^ = **D** ^(4)^. We have mentioned at the beginning of this section that the composite laminate has a symmetrically located delamination at its center, in geometrical expressions *l*_1_ = *l*_4_, *l*_2_ = *l*_3_. Under such circumstance we have *λ*_*G*_^(*1*)^ = *λ*_*G*_^(*4*)^ from Eqs (34) and (36). It illuminates that the modal parameters of the global vibration for Sub-laminates *Ω* ^(*1*)^ and *Ω* ^(*4*)^ are the same. So do those for Sub-laminates *Ω* ^(2)^ and *Ω* ^(3)^, as shown in Eq. (35). We now observe that the coefficient constants of the global vibrations for Sub-laminates *Ω* ^(*2*)^ and *Ω* ^(*3*)^ are almost equal to each other, as given in the first of Eq. (37). All the relationships rationalize that Sub-laminate *Ω* ^(*2*)^ and Sub-laminate *Ω* ^(*3*)^ exhibit an identical global vibration. This holds for Sub-laminate *Ω* ^(*1*)^ and Sub-laminate *Ω* ^(*4*)^ as well because of symmetrical delamination.

Based on above observation, we estimate the approximate values of *λ*_*G*_^(*i*)^, the global mode parameters, and *λ*_*L*_, the local mode parameter. Normally, the geometries of the composite laminates have multiple scale features: m-lever length *l*_*i*_ and width *b* of Sub-laminate *Ω* ^(*i*)^, cm-lever thickness *h*_*i*_ of Sub-laminate *Ω* ^(*i*)^, and mm-lever thickness of a single composite ply *h*_*0*_. For laminates of such geometries, the nondimensional quantities *a*_*i*_, *b*_*i*_ and *c*_*i*_ in Eq. (34) are estimated to be proportional to *K*_*i*_^2^(*h*_0_/*l*_*i*_)^4^ in which *K*_*i*_ is the number of composite ply of Sub-laminate *Ω* ^(*i*)^, therefore they are roughly of the same magnitude order. Depending on the geometries of a particular delamination, the magnitudes of *a*_*i*_, *b*_*i*_ and *c*_*i*_ range from 10^−12^ to 10^−7^. On the other hand, the nondimensional interaction factor $\bar{k}$ for composite laminate made of graphite/epoxy composite plies with defined material properties in the first paragraph of this section is approximated to be 10^−4^. With such properties, the numerical values of global vibration *λ*_*G*_^(*i*)^ and local buckling *λ*_*L*_ in Eq. (35) are approximated to be *λ*_*G*_^(*i*)^ ∝ *b*_*i*_/*a*_*i*_ and *λ*_*L*_ ∝ ($\bar{k}$/*a*_2_)^1/4^, respectively. Considering that *a*_*i*_, *b*_*i*_, *c*_*i*_ ∝ *K*_*i*_^2^(*h*_0_/*l*_*i*_)^4^ and $\bar{k}$ ≫ *a*_2_, it is easy to see that *λ*_*L*_ ≫ *λ*_*G*_^(*i*)^. Our further investigations indicate (except very limit conditions when *l*_*i*_ < 0.05*a*) that

39 $$0 < {\lambda }_{G}^{(i)} < 2\quad \quad {\rm{and}}\quad \quad {\lambda }_{L} > 10{\lambda }_{G}^{(i)}$$

As the global vibration and local buckling are the effective components of the total free vibration mode, their leading order terms in Eq. (33) must be of the same order, i.e. ${G}_{1}^{(i)}{e}^{{\lambda }_{G1}^{(i)}}\propto {L}_{1}^{(i)}{e}^{{\lambda }_{L}}$. Using Eq. (39), the following relation is established

40 $${G}_{1}^{(i)}\propto {10}^{3}{L}_{1}^{(i)}.$$

Equation (40) illustrates that the amplitudes of global vibration are about 10^3^ times of those of local buckling, that is to say, the global vibration is macroscopic and the local buckling is microscopic. In addition, it can be seen from Eqs (34–36) that the global vibration modes *λ*_*G*_^(*i*)^ and the local buckling mode *λ*_*L*_ are related to the nondimensional frequency $\bar{\omega }$ and the nondimensional interaction factor $\bar{k}$, respectively. Referring to Eqs (11), (23) and (35), it is found that the value of *λ*_*L*_ is determinate for a particular case in which material properties and geometric parameters of the laminate and the delamination are all known. Determination of ^*λ*^_*G*_^(*i*)^ calls for the application of the equilibrium conditions and the continuity conditions in Eqs (27–29) and the boundary conditions in Eq. (30) from which a set of homogeneous equations of the indeterminate coefficients are derived and are expressed in a concise manner as follows:

41 $$[R(\bar{\omega })][\bar{{\rm{{\rm X}}}}]=0,$$

where $[R(\bar{\omega })]$ is 16-by-16 coefficients matrix containing the nondimensional frequency $\bar{\omega }$ as the only unknown and $[\bar{{\rm{{\rm X}}}}]$ is a collection of the sixteen indeterminate coefficients given by

42 $$[\bar{{\rm X}}]={[{G}_{1}^{(1)}\mathrm{...}{G}_{4}^{(1)},{G}_{1}^{(2)}\mathrm{...}{G}_{4}^{(2)},{L}_{1}^{(2)}\mathrm{...}{L}_{4}^{(2)},{G}_{1}^{(4)}\mathrm{...}{G}_{4}^{(4)}]}^{T}.$$

The premise of solving the homogeneous Eq. (41) is the determinant of $[R(\bar{\omega })]$ to be zero, i.e.

43 $$|R(\bar{\omega })|=0.$$

Equation (43) is the eigen function for the nonlinear free vibration of composite laminate containing an embedded delaminate in which the nondimensional natural frequency of the delaminated composite laminate $\bar{\omega }$ is available.

## Results

In order to study the performance of the nonlinear vibration of the delaminated laminates, we conduct numerical analysis. Based on the results presented above, we develop MATLAB programs to perform numerical calculations. In the implementations, the laminate has a geometry size of 2 m × 2 m × 0.12 m and is composed of 60 plies made of T300/QY8911 carbon fiber reinforced composite material with the density *ρ* = 2150 kg/m^3^ stacked in a sequence of [0°/90°/0°]_20_ with each single-layer thickness *h*_0_ = 0.002 m. The equivalent material properties of each composite ply are still the same as those in Nonlinear dynamic stability analysis, i.e. *E*_11_ = 140 GPa, *E*_22_ = 10 GPa, *G*_12_ = 5 GPa, and *μ*_12_ = 0.3.

**Figure 3:**
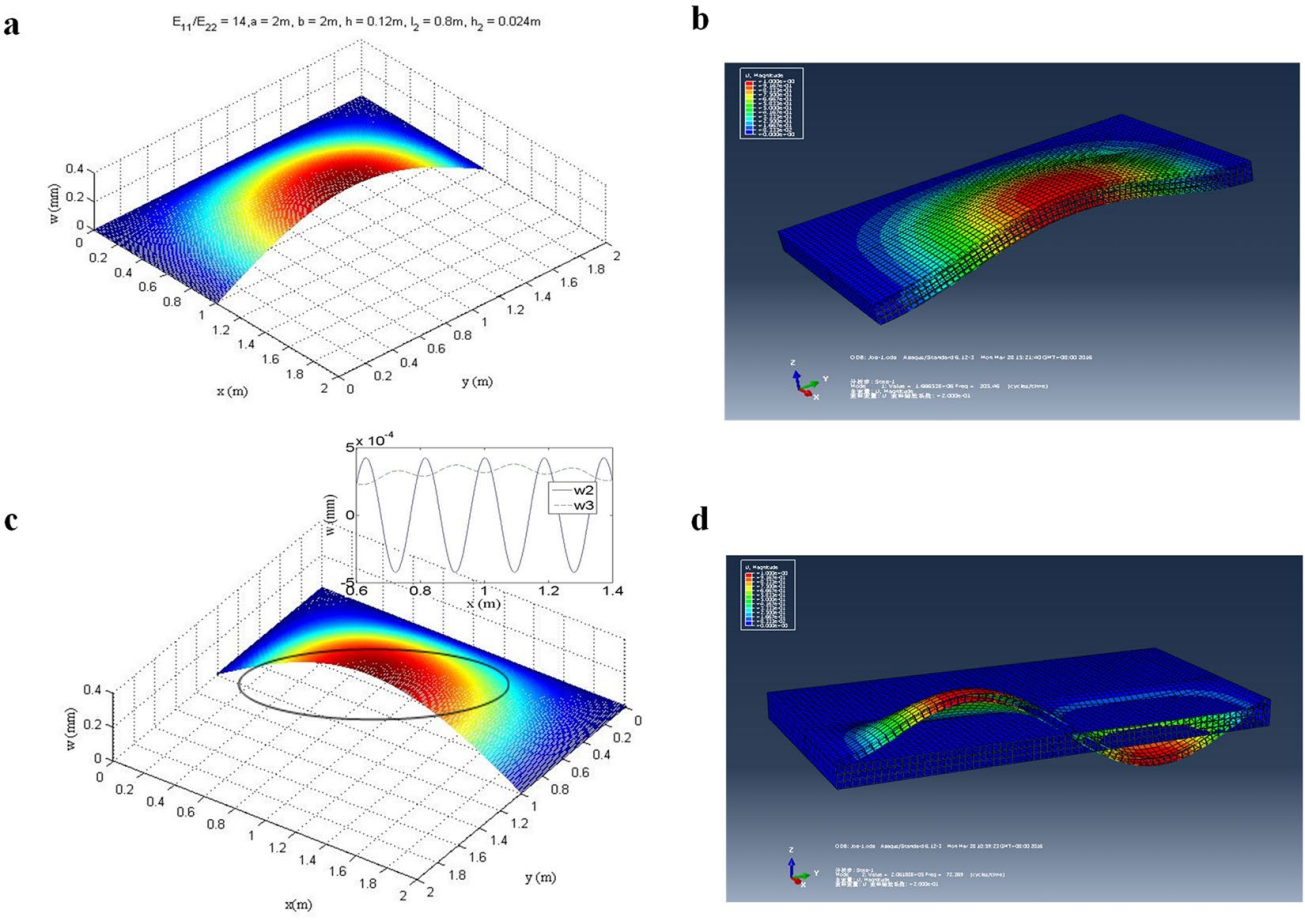
Free vibration modes for *η* = 0.5 and *τ* = 0.3. (**a**) Profile of analytical vibration mode at cross-section *x* = *a*/2. (**b**) Profile of vibration mode at cross-section *x* = *a*/2 from ABAQUS with interaction penalty. (**c**) Profile of vibration mode with local buckling superposed. (**d**) Vibration mode from ABAQUS without interaction penalty.

The data obtained enable us to clearly reveal the nature of the free vibration of composite laminates containing a buried delamination. The profile of the vibration modes of the delaminated laminate at the cross section of *x* = *a*/2 is shown in Fig. 3a obtained from MATLAB programs for analytical solution given the non-dimensional delamination length *η* = *l*_2_/*l* = 0.5 and the non-dimensional delamination depth *τ* = *h*_2_/*h* = 0.3. As a comparison, we also perform finite element simulation using ABAQUS software. Unlike the shell theory in theoretical analysis, we use solid elements and General Contact Interaction Module in ABAQUS analysis in order to incorporate the anti-penetration penalty at the interfaces of sublaminates *Ω* ^(2)^ and *Ω* ^(3)^–above and below the delamination–to prevent them from penetrating with each other during vibration. Figure 3b is the free vibration of the delaminated laminate from ABAQUS analysis for the same *η* and *τ* used in Fig. 3a. The profile of the vibration mode in Fig. 3b makes it clear to us that the delaminated composite laminate vibrates as a single whole–sublaminates above and below the delamination vibrate identically. This is exactly the same as what we predict in theoretical analysis given in Fig. 3a. The observation of consistent global vibration mode in ABAQUS justifies our analytical solutions and makes our analysis effective.

We have discussed that except the consistent global vibration the nonlinear vibration of the delaminated composite laminate includes a local buckling. Figure 3c shows the buckling modes in *x*-direction at cross section *y* = 1 m for both Sublaminates *Ω* ^(2)^ and *Ω* ^(3)^. Distinct from their consistent global vibrations, their local buckling modes of Sublaminates *Ω* ^(2)^ and *Ω* ^(3)^ are not identical—not only the buckling amplitude of Sublaminates *Ω* ^(2)^ is much larger than that of Sublaminate *Ω* ^(3)^ due to its weaker stiffness, but also their amplitudes are completely reversal with each other. It is also found from Fig. 3c that the two sublaminates buckle in modes of equal but quite short wavelength. With these features, once the delaminated laminate undergoes vibration, local voids are formed on the interfaces at places where upper Sublaminate *Ω* ^(2)^ and lower Sublaminate *Ω* ^(3)^ buckles upwards and downwards respectively. Between the voids the two sublaminates buckle in the opposite directions. Interaction forces are produced in these regions. With these characteristics, voids and interaction areas are developed alternately at the interfaces of the delamination, as illustrated in Fig. 3c. It is remarkable that the magnitudes of the local buckles are only about 10^−3^ of the magnitudes of the vibration modes, so the voids are microscopic, invisible to the naked eyes.

**Figure 4:**
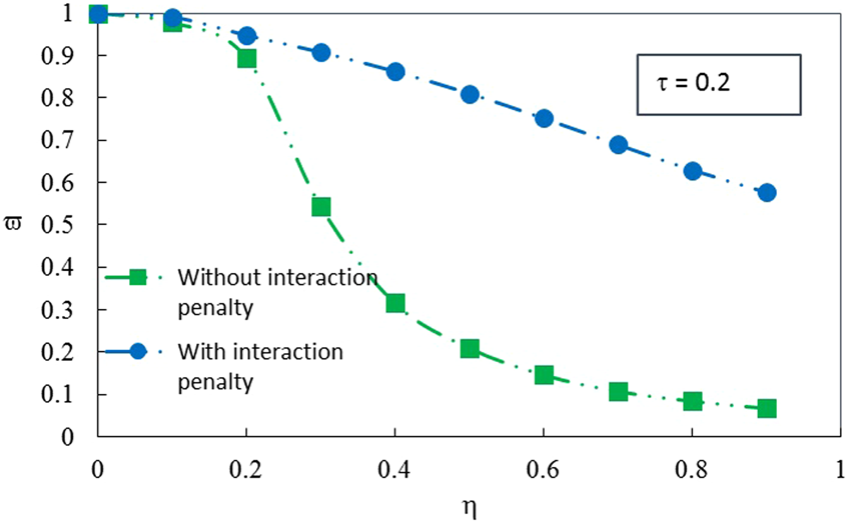
A comparison of the free vibration frequency of the delaminated laminate with/without interaction penalty at the interfaces of the delamination from finite element analysis.

For a more comprehensive understanding of the situation, we further investigate cases of different length of delamination with/without the interaction penalty. Figure 4 shows a comparison of the free vibration frequency of the delaminated laminate with/without interaction penalty enforced at the interfaces of the delamination from ABAQUS analysis. The results reveal that the delaminated laminate vibrates in modes of much high frequency when the penalty conditions are enforced. The reason for such difference is that the penalty conditions link the separate sublaminates together as a unitary one, thus greatly enhance the stiffness of the delaminated laminate.

**Figure 5:**
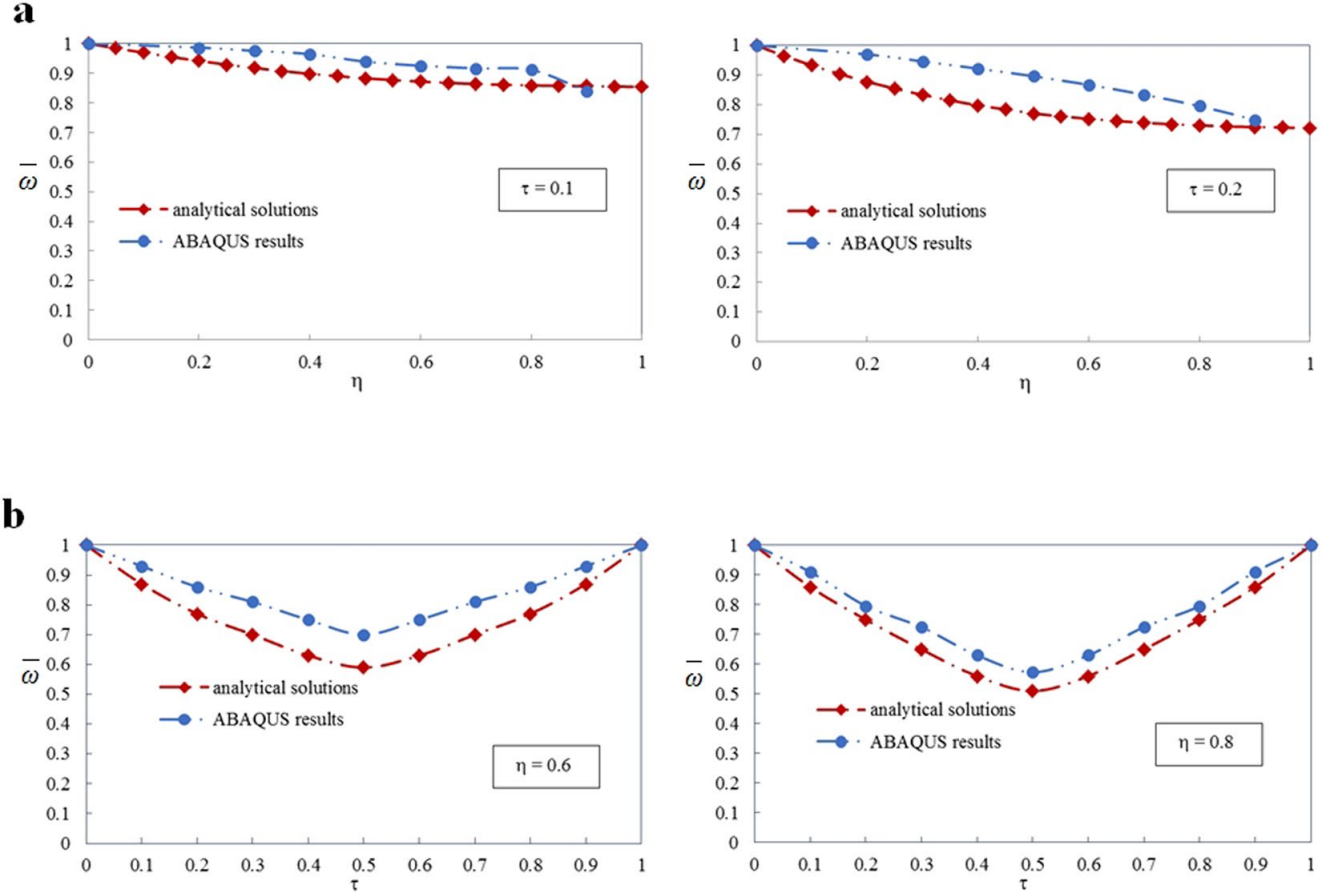
Influence of the geometric parameters of the delamination on the vibration frequency. (**a**) Variation of the nondimensional frequency $\bar{\omega }$ with respect to the nondimensional delamination length *η* for the nondimensional delamination depth *τ* = 0.1 and *τ* = 0.2. (**b**) Variation of the nondimensional frequency $\bar{\omega }$ with respect to the nondimensional delamination depth *τ* for the nondimensional delamination length *η* = 0.6 and *η* = 0.8.

We further carry out a parametric study to investigate the influence of the delamination properties on the vibration frequency. Variation of the nondimensional frequency $\bar{\omega }$ with respect to the nondimensional delamination length *η* are given in Fig. 5a given the nondimensional delamination depth τ = 0.1 and τ = 0.2. As a comparison, the results from ABAQUS analysis are drawn in the diagram too. Both the analytical solutions and ABAQUS results show that the nondimensional frequency $\bar{\omega }$ decreases with the increasing of the nondimensional delamination length *η*. It is a common sense that the smaller the stiffness of a laminate, the larger its amplitude and the lower its frequency. The delaminated laminate is actually a single whole with reduced stiffness due to the existence of the delamination. As the length of the delamination enlarges, the stiffness of the laminate continues deteriorating, thus leading to the decreasing of the frequency.

We also plot variation of the nondimensional frequency $\bar{\omega }$ with respect to the nondimensional delamination depth τ in Fig. 5b given the nondimensional delamination length *η* = 0.6 and *η* = 0.8 for both analytical solutions and ABAQUS results. Likewise, the nondimensional frequency $\bar{\omega }$ reduces as the value of nondimensional delamination depth τ rises. Such trend is still owing to the degradation of the stiffness. We are clear that the stiffness of the delaminated laminate depends on the stiffness and the geometries of the four sublaminates. For a specific length of the delamination, the geometries of the four sublaminates as well as the stiffness of Sublaminates *Ω* ^(1)^ and *Ω* ^(4)^ are invariable. Under such circumstance, the stiffness of the delaminated laminate is determined either by Sublaminate *Ω* ^(2)^ or Sublaminate *Ω* ^(3)^, whichever has a larger stiffness. As the delamination moves from the surface to the middle of the laminate, the stiffness of Sublaminate *Ω* ^(3)^ continues to decrease but still exceeds that of Sublaminate *Ω* ^(2)^, thus the stiffness of the delaminated laminate is continuously reduced. At the middle plane, Sublaminates *Ω* ^(2)^ and *Ω* ^(3)^ possess equal stiffness and the delaminated laminate has the lowest stiffness. Once the delamination is in the lower part of the laminate, the stiffness of the delaminated laminate is mainly controlled by Sublaminates *Ω* ^(2)^ instead of *Ω* ^(3)^ and increases with the delamination depth.

Comparison of the nondimensional frequency $\bar{\omega }$ with ABAQUS results is presented in Fig. 5b, too. One can see from Fig. 5 that the results from finite element simulation and theoretical analysis have some deviations. The nondimensional frequencies $\bar{\omega }$ from ABAQUS simulation are numerically slightly larger than the analytical predictions. Besides, the declining trends of $\bar{\omega }$ with respect to *η* from the two approaches are somewhat different, as shown in Fig. 5a. These deviations are due to the facts that we use the two-dimensional plate and shell theory to carry on the theoretical analysis but a three-dimensional solid model to carry on the finite element analysis. To prevent the penetration between sublaminates *Ω* ^(2)^ and *Ω* ^(3)^ in ABAQUS simulation, hard contact property is defined in interaction module to impose constrains on the bottom of *Ω* ^(2)^ and on the top of *Ω* ^(3)^. However such application is practicable only to solid model in which shear modulus in transverse direction are necessary. As the shear deformation in transverse direction is omitted in theoretical analysis, inevitably, there are deviations between the theoretical and finite element results. Nevertheless, the comparisons are still adequate in validating our theoretical predictions.

## Discussion

In summary, we present an analysis of the nonlinear vibration of composite laminates containing a buried delamination through the newly developed multiscale framework. The delaminated laminate is treated as four independent sublaminates with granted restrains of forces and deformations at the delamination fronts to reunite them as single whole. By incorporating the anti-penetrating interaction kinematics at the interfaces of the delamination into the nonlinear theory for vibration of composite laminate, the nonlinear vibration of delaminated composite laminates is explored. We consider the multiscale compositions of the laminate and identify two failure modes of global vibration and local buckling. The modes of the global vibration are macroscopic and compatible among the four sublaminates with exactly the same frequency. In particular, sublaminates above and below the delamination undergoes a fully consistency vibration. On the other hand, the local buckling is microscopic, occurring only between sublaminates above and below the delamination with completely reverse amplitudes that are about 10^−3^ times smaller than those of the vibration mode. By developing numerical MATLAB program, we investigate the effects of the delamination on the intrinsic behaviors of composite laminate over a wide range of the delamination length and the delamination depth. The free vibration frequency is shown to monotonic decrease upon increasing of the delamination length and to decrease then increase as the delamination depth increases. We perform finite element simulations for the free vibration of delaminated laminates with/without interaction penalty enforced at the interfaces of the delamination, which suggest that the interaction penalty influence not only the vibration frequency but also the vibration mode. For laminates of the same geometry and delamination parameters, the one without the interaction penalty has much lower vibration frequency than that with the interaction penalty. The difference is reflected on the vibration mode in which sublaminate above and below the delamination penetrate with each other when the interaction penalty are not enforced. The characteristic behaviors of the delaminated laminate are consistent in both theoretical solutions and finite element results, thereby demonstrating the effective of the analysis.

## Methods

### Separation of variable

To solve the dimensionless formulas of Eqs (24–26), we use the method of separation of variables by taking the dimensionless deflection as follows:

44 $${\bar{w}}^{(i)}(\bar{x},\bar{y},\bar{t})=(A\,\cos \,\bar{t}+B\,\sin \,\bar{t}){\bar{W}}^{(i)}(\bar{x},\bar{y})$$

where ${\bar{W}}^{(i)}(\bar{x},\bar{y})$ is the dimensionless free vibration mode of Sublaminate *Ω* ^(*i*)^ and must satisfy the boundary conditions of Sublaminate *Ω* ^(*i*)^. Noticing that each of Sublaminate *Ω* ^(*i*)^ is just only simply supported in *y* direction, the free vibration mode ${\bar{W}}^{(i)}(\bar{x},\bar{y})$ is chosen as the following form so as to satisfy the boundary conditions of Eq. (31)^38^

45 $${\bar{W}}^{(i)}(\bar{x},\bar{y})={f}^{(i)}(\bar{x})\cdot \,\sin (\pi \bar{y})$$

Put Eq. (45) into (44) and then substitute the acquired ${\bar{w}}^{(i)}(\bar{x},\bar{y},\bar{t})$ into Eqs (24–26). Decoupling ${f}^{(2)}(\bar{x})$ and ${f}^{(3)}(\bar{x})$ from the acquired equations we derive the following set of ordinary differential equations of ${f}^{(i)}(\bar{x})$

46 $${a}_{i}\frac{{d}^{4}{f}^{(i)}(\bar{x})}{d{\bar{x}}^{4}}-{b}_{i}\frac{{d}^{2}{f}^{(i)}(\bar{x})}{d{\bar{x}}^{2}}+{c}_{i}{f}^{(i)}(\bar{x})=0\quad \quad ({\rm{for}}\,{\Omega }^{(1)}{\&}{\Omega }^{(4)}),$$

47 $$\begin{array}{l}{a}_{3}{a}_{2}\frac{{d}^{8}{f}^{(2)}(\bar{x})}{d{\bar{x}}^{8}}-({a}_{3}{b}_{2}+{b}_{3}{a}_{2})\frac{{d}^{6}{f}^{(2)}(\bar{x})}{d{\bar{x}}^{6}}+[{a}_{3}({c}_{2}-k)+{b}_{3}{b}_{2}+{a}_{2}({c}_{3}-k)]\frac{{d}^{4}{f}^{(2)}(\bar{x})}{d{\bar{x}}^{4}}\\ \quad \,\,-[{b}_{3}({c}_{2}-k)+{b}_{2}({c}_{3}-k)]\frac{{d}^{2}{f}^{(2)}(\bar{x})}{d{\bar{x}}^{2}}+[{c}_{3}{c}_{2}-{c}_{3}k-{c}_{2}k]{f}^{(2)}(\bar{x})=0\end{array}\quad (\mathrm{for}\,{\Omega }^{(2)}),$$

48 $${f}^{(3)}(\bar{x})=-\frac{{a}_{2}}{\overline{k}}\frac{{d}^{4}{f}^{(2)}(\bar{x})}{d{\bar{x}}^{4}}+\frac{{b}_{2}}{\overline{k}}\frac{{d}^{2}{f}^{(2)}(\bar{x})}{d{\bar{x}}^{2}}-\frac{{c}_{2}}{\overline{k}}{f}^{(2)}(\bar{x})+{f}^{(2)}(\bar{x})\quad \quad (\mathrm{for}\,{\Omega }^{(3)})$$

The values of *a*_*i*_, *b*_*i*_ and *c*_*i*_ in above equation are given in Eq. (36). To solve Eqs (46) and (47), we take exponential function ${f}^{(i)}(\bar{x})={e}^{{\lambda }^{(i)}\bar{x}}$ and obtain a set of equations of the eigenvalue *λ* ^(*i*)^ which is given below in order to facilitate the analysis in detail

49 $${a}_{i}{({\lambda }^{(i)})}^{4}-{b}_{i}{({\lambda }^{(i)})}^{2}+{c}_{i}=0\quad \quad ({\rm{for}}\,{\Omega }^{(1)}{\&}{\Omega }^{(4)})$$

50 $${({\lambda }^{(2)})}^{8}+{\xi }_{1}{({\lambda }^{(2)})}^{6}+{\xi }_{2}{({\lambda }^{(2)})}^{4}+{\xi }_{3}{({\lambda }^{(2)})}^{2}+{\xi }_{4}=0\quad \quad ({\rm{for}}\,{\Omega }^{(2)}{\&}{\Omega }^{(3)})$$

where

51 $$\begin{array}{rcl}{\xi }_{1} & = & -({b}_{2}{a}_{3}+{a}_{2}{b}_{3})/{a}_{2}{a}_{3}\\ {\xi }_{3} & = & -({c}_{2}{b}_{3}+{b}_{2}{c}_{3}-{b}_{2}\overline{k}-{b}_{3}\overline{k})/{a}_{2}{a}_{3}\end{array}\quad \begin{array}{rcl}{\xi }_{2} & = & ({c}_{2}{a}_{3}+{b}_{2}{b}_{3}+{a}_{2}{c}_{3}-{a}_{2}\overline{k}-{a}_{3}\overline{k})/{a}_{2}{a}_{3}\\ {\xi }_{4} & = & ({c}_{2}{c}_{3}-{c}_{2}\overline{k}-{c}_{3}\overline{k})/{a}_{2}{a}_{3}\end{array}$$

### Multiscale analysis

Solutions of *λ* ^(1)^ and *λ* ^(4)^ in Eq. (49) for Sublaminates *Ω* ^(2)^ and *Ω* ^(3)^ are quite clear and are given in Eq. (34). As for those of Eq. (50), they are not straightforward. We have pointed out in sub section Nonlinear dynamic stability analysis that due to the multiscale feature of the laminate composition, the magnitudes of *a*_*i*_, *b*_*i*_, *c*_*i*_ are of the same order, approximately ranging from 10^−12^ to 10^−7^ for a typical delamination. While the nondimensional contact factor $\overline{k}$ has a magnitude of about 10^−4^ for T300/QY8911 carbon fiber reinforced composite laminates. Therefore, the coefficients *ξ*_1_ ~ *ξ*_4_ in Eq. (51) have approximate magnitudes as follows:

52 $${\xi }_{1}\propto {10}^{0},\quad {\xi }_{2}\propto (-{a}_{2}\overline{k}-{a}_{3}\overline{k})/{a}_{2}{a}_{3},\quad {\xi }_{3}\propto -(-{b}_{2}\overline{k}-{b}_{3}\overline{k})/{a}_{2}{a}_{3},\quad {\xi }_{4}\propto (-{c}_{2}\overline{k}-{c}_{3}\overline{k})/{a}_{2}{a}_{3}$$

Considering the magnitudes of *a*_*i*_, *b*_*i*_, *c*_*i*_ and $\overline{k}$, we found that *ξ*_2_, *ξ*_3_, *ξ*_4_ > 10^4^ ≫ *ξ*_1_ < 2. With such coefficient properties, Eq. (52) must have both small roots and large roots, i.e., a multiscale features. The small roots correspond to *ξ*_1_ of magnitude 1, the large ones correspond to *ξ*_2_, *ξ*_3_, *ξ*_4_. We decompose Eq. (50) into the following form:

53 $$[{({\lambda }^{(2)})}^{4}+{\phi }_{1}{({\lambda }^{(2)})}^{2}+{\phi }_{2}]\cdot [{({\lambda }^{(2)})}^{4}+{\phi }_{3}]=0,$$

where *φ*_1_ and *φ*_2_ are undetermined coefficients corresponding to small roots of order 1 and *φ*_3_ corresponding to large roots with a numerical value larger than 10^4^. Expand Eq. (53) and compare with the same term in Eq. (52). The coefficients *φ*_1_, *φ*_2_ and *φ*_3_ are found

54 $${\phi }_{1}\approx -\frac{({b}_{2}+{b}_{3})}{({a}_{2}+{a}_{3})},\quad \quad {\phi }_{2}\approx \frac{({c}_{2}+{c}_{3})}{({a}_{2}+{a}_{3})},\quad \quad {\phi }_{3}\approx -\frac{({a}_{2}+{a}_{3})\bar{k}}{({a}_{2}\cdot {a}_{3})}\cdot $$

Now Eq. (53) can be easily solved. The multiscale solutions of *λ* ^(2)^ are given in Eq. (35). With the available solutions of *λ* ^(2)^, we can formulate the vibration mode in *x* direction${f}^{(2)}(\bar{x})$ of Sublaminate *Ω* ^(2)^

55 $$\begin{array}{rcl}{f}^{(2)}(\bar{x}) & = & \{[{G}_{1}^{(2)}{e}^{{\lambda }_{G1}^{(2)}\bar{x}}+{G}_{2}^{(2)}{e}^{-{\lambda }_{G1}^{(2)}\bar{x}}+{G}_{3}^{(2)}\,\cos ({\lambda }_{G2}^{(2)}\bar{x})+{G}_{4}^{(2)}\,\sin ({\lambda }_{G2}^{(2)}\bar{x})]\\  &  & +[{L}_{1}^{(2)}{e}^{{\lambda }_{L}\bar{x}}+{L}_{2}^{(2)}{e}^{-{\lambda }_{L}\bar{x}}+{L}_{3}^{(2)}\,\cos ({\lambda }_{L}\bar{x})+{L}_{4}^{(2)}\,\sin ({\lambda }_{L}\bar{x})]\}\,for\,{{\rm{\Omega }}}^{(2)}\end{array}$$

Substituting the obtained ${f}^{(2)}(\bar{x})$ into Eq. (48), we can determine the solution of ${f}^{(3)}(\bar{x})$: it is quite similar to Eq. (55) and has a close relation with solution of ${f}^{(2)}(\bar{x})$: *λ* ^(3)^ = *λ* ^(2)^ (see Eq. (35)) and *G* ^(3)^ ≈ *G* ^(2)^ and *L* ^(3)^ = −*ιL* ^(3)^ (see Eqs (37) and (38)).

Replacing ${f}^{(2)}(\bar{x})$ and ${f}^{(3)}(\bar{x})$ into Eq. (45) and then into (44), we obtain the solutions of vibration mode for Sublaminate *Ω* ^(2)^ and Sublaminate *Ω* ^(3)^ which is expressed as a concise formula in Eq. (33).
